# Intestinal epithelial *C/EBPβ* deficiency impairs colitis-associated tumorigenesis by disrupting CXCL1/CXCL2/CXCL5-CXCR2-mediated neutrophil infiltration: Epithelial C/EBPβ loss suppresses colitis-associated cancer

**DOI:** 10.3724/abbs.2025119

**Published:** 2025-09-08

**Authors:** Mingyue Li, Xintong Wang, Wenjie Hu, Xiaohui Cheng, Qi Sun, Yongjie Wu, Zhen Huang, Jiangning Chen

**Affiliations:** 1 State Key Laboratory of Analytical Chemistry for Life Sciences School of Life Sciences Nanjing University Nanjing 210023 China; 2 State Key Laboratory of Pharmaceutical Biotechnology School of Life Sciences Nanjing University Nanjing 210023 China; 3 NJU Xishan Institute of Applied Biotechnology Wuxi 214101 China

**Keywords:** colitis-associated tumorigenesis, epithelial-specific C/EBPβ, CXCL1/CXCL2/CXCL5-CXCR2 axis, CXCR2-mediated neutrophil infiltration

## Abstract

Dysregulated transcription factors critically link chronic inflammation to oncogenesis in colitis-associated colorectal cancer (CAC), but their mechanistic roles remain incompletely understood. By integrating microarray and transcriptome sequencing data from ulcerative colitis (UC), colitis-associated cancer (CAC), and colorectal cancer (CRC) patients, we identify C/EBPβ as a key transcriptional regulator whose elevated expression inversely correlates with survival. In azoxymethane (AOM)/dextran sulfate sodium (DSS)-induced CAC models, intestinal epithelial C/EBPβ is upregulated during tumor progression, which is correlated with exacerbated tumor burden and neutrophil infiltration. Mice with intestinal epithelial-specific *Cebpb* deletion ( *Cebpb* ^ΔIEC^) are resistant to carcinogenesis, accompanied by reduced neutrophil infiltration and tumor growth. Mechanistically, C/EBPβ transcriptionally activates CXCR2 ligands (CXCL1, CXCL2, and CXCL5) to drive neutrophil recruitment. Pharmacological inhibition of CXCR2 phenocopies the anti-tumor effects of *Cebpb* ^ΔIEC^ deletion, further validating this axis as a therapeutic target. Correlation analysis of patient tissues confirms positive relationships between C/EBPβ, CXCR2 ligands, and neutrophil infiltration, suggesting that targeting the C/EBPβ-CXCL1/2/5-CXCR2 axis may constitute a novel strategy for treating CAC.

## Introduction

Chronic inflammation is a well-established driver of carcinogenesis that significantly contributes to the global disease burden and mortality [ 1, 2]. This relationship is particularly evident in colorectal cancer (CRC), the third leading cause of cancer mortality worldwide [3], where inflammation-mediated oncogenesis plays a crucial role [ 4, 5]. The connection is most pronounced in colitis-associated colorectal cancer (CAC), which develops in 2.4% of inflammatory bowel disease (IBD) patients within 20 years of diagnosis [6]. Notably, only 5.2% (95% CI 4.3%–6.1%) of CRC cases originate from overt inflammation [ 7, 8]. These patients exhibit markedly worse 5-year survival rates than sporadic CRC patients do (18.3% vs 64.1%, *P <*0.001) [9], primarily because of therapeutic resistance [10]. The molecular pathogenesis of CAC involves multiple mechanisms: (i) early activation of oncogenic pathways, as evidenced by the 3.2-fold higher frequency of KRAS ^G12D^ mutations than in sporadic CRC [11]; (ii) dysregulation of key tumor suppressors, including TP53 and DCC [ 12– 14]; and (iii) establishment of a persistent inflammatory microenvironment. Despite these advances in understanding CAC development, the precise mechanistic links between chronic inflammation and CAC progression remain incompletely characterized, particularly with respect to the transcriptional regulation of this process.

CAC pathogenesis involves transcriptional reprogramming that links inflammation to malignancy. Central to this process are transcription factors that coordinately regulate gene networks governing immune homeostasis and cellular proliferation. Key players include early pathogenic mediators such as *TP53* mutations and constitutive TNF/NF-κB activation [ 15, 16], along with dysregulated cofactors such as NFKBIZ [ 17 , 18] and epithelial-specific defects such as SMAD4 loss (elevating CCL20-CCR6 axis activity) [19] and ITF2 deficiency (promoting p65 nuclear translocation) [17]. Among the transcriptional regulators linking inflammation to carcinogenesis, CCAAT/enhancer-binding protein β (C/EBPβ) has emerged as a critical mediator. This conserved transcription factor contains a basic leucine zipper (bZIP) domain enabling DNA binding through homo- or heterodimerization [20] and gives rise to three functionally distinct isoforms: liver enriched activator protein (LAP1/LAP2) and liver enriched inhibitory protein (LIP) [21]. The activity of C/EBPβ is finely tuned through post-translational modifications that regulate its subcellular localization and protein interactions. Physiologically, C/EBPβ orchestrates diverse programs ranging from cell proliferation and differentiation to immune responses [22], while pathologically, it integrates key inflammatory signals, including the MAPK/NF-κB and JAK/STAT3 pathways [23]. Our previous study demonstrated its oncogenic role through the C/EBPβ-miR-223-RASA1 axis in colorectal cancer [24]. Notably, despite these advances in understanding the general functions of C/EBPβ, its epithelium-specific mechanisms in CAC pathogenesis, particularly regarding neutrophil recruitment through chemokine regulation, remain poorly characterized.

In the present study, our multi-omics analysis of ulcerative colitis (UC), CRC, and CAC patient datasets identified C/EBPβ as a key transcription factor in CAC pathogenesis. Clinical samples and azoxymethane (AOM)/dextran sulfate sodium (DSS)-induced mouse models confirmed that intestinal epithelial C/EBPβ drives CAC progression, with genetic deletion reducing inflammation and tumor burden. Mechanistically, C/EBPβ promotes neutrophil recruitment through the transcriptional activation of CXCR2 ligands (CXCL1/2/5), thereby establishing a pro-tumorigenic inflammatory microenvironment. CXCR2 inhibition attenuated neutrophil infiltration, and human tissue analyses confirmed C/EBPβ-CXCR2 ligand correlations. Our study identifies epithelial C/EBPβ as a previously unrecognized mechanistic link connecting inflammation to CAC progression, specifically via transcriptional activation of CXCR2 ligands. The C/EBPβ-CXCL1/2/5-CXCR2 axis represents a novel therapeutic target for preventing inflammation-driven colorectal carcinogenesis.

## Materials and Methods

### Clinical samples

Clinical samples of UC, CAC and CRC patients from public datasets (GEO: GSE87466, GSE37283; TCGA-COAD/READ) were analyzed ( Supplementary Tables S1–S3). Human tissue samples were analyzed from: (1) 180 CRC patient-matched pairs (Outdo Biotech, Shanghai, China; Supplementary Table S4), (2) 5 UC patients and 5 CAC patients with matched controls plus 10 normal biopsies (Nanjing Jinling Hospital, Nanjing, China; Supplementary Table S5), and (3) 64 additional CRC patients (Nanjing Jinling Hospital; Supplementary Table S6). The study was conducted with ethics approval (Jinling Hospital; No. 2021DZSKT-YBB-008) and informed consent.

### Reagents

Reagents included AOM and SB225002 (Sigma, St Louis, USA), DSS (MP Biomedicals, Solon, USA), *CEBPB* siRNA from Thermo Fisher Scientific (Waltham, USA) ( Supplementary Table S7), and a pcDNA3.1-CEBPB plasmid (Real Gene, Nanjing, China). The luciferase reporters (pGL3 vectors; Promega, Madison, USA) contained 2 kb promoters of CXCL1/2/5 with wild-type or mutated *CEBPB* sites. For CXCL1, the sequence CTTTCAAAAT was altered to ACGGTCCCCA; for CXCL2, GTTTCACAAC was changed to AGGGTCTCCT; and for CXCL5, TCTTGCTCCAT was replaced by TCAATTTTTAT. Biotin-labelled and unlabelled probes were synthesized by GeneBio Co., Ltd (Shanghai, China; Supplementary Table S7). Recombinant human C/EBPβ protein was purchased from Proteintech (Wuhan, China).

### Animal studies

Male C57BL/6J mice (6–8 weeks old) were purchased from GemPharmatech (Nanjing, China). Intestinal epithelial-specific *Cebpb*-knockout ( *Cebpb* ^ΔIEC^) mice were generated by crossing B6.129S1- *Cebpb* ^tm1Rcsm^/Mmnc mice with B6. Cg-Tg (Vil1-cre) 1000Gum/J strains. The mice were housed under SPF conditions. All animal experiment procedures were approved by the Nanjing University Animal Ethics Committee (IACUC-2006015).

For CAC induction, 8-week-old mice received 10 mg/kg AOM intraperitoneally, followed by three cycles of 2.5% DSS (1 week) and normal water (2 weeks). SB225002 (10 mg/kg, twice weekly) was used to assess neutrophil infiltration. The mice were randomized into five groups ( *n* = 5/group): (1) wild-type (WT) controls receiving intraperitoneal saline and normal drinking water; (2) *Cebpb* ^ΔIEC^ mice treated with AOM/DSS; (3) *Cebpb* ^ΔIEC^ mice treated with AOM/DSS/SB225002; (4) WT mice treated with AOM/DSS; and (5) WT mice treated with AOM/DSS/SB225002. On day 63, the colons were collected for histopathology, qRT-PCR, western blot analysis, flow cytometry, RNA-seq, and ELISA.

### Histopathological analysis

Human and mouse tissues were fixed, paraffin-embedded and sectioned. H&E staining was performed according to the manufacturer’s protocols (Leagene Biotechnology, Beijing, China), with mouse tumors classified as low-grade dysplasia, high-grade dysplasia, or invasive carcinoma using established criteria [25]. For immunofluorescence, antigen-retrieved sections were incubated overnight at 4°C with Alexa Fluor-conjugated antibodies (Ly-6G, CD11b), followed by nuclear counterstaining with DAPI (Beyotime, Shanghai, China), with appropriate isotype controls. Images were acquired using a confocal microscope (LSM 980; ZEISS, Wetzlar, Germany). Immunohistochemical staining involved overnight incubation at 4°C with primary antibodies (C/EBPβ, CXCL1/2/5 and CD66b) after antigen retrieval and peroxidase blocking, followed by 45-min incubation at room temperature with HRP-conjugated secondary antibodies, DAB development, and hematoxylin counterstaining, with imaging performed on an Eclipse Ni-E upright microscope (Nikon, Tokyo, Japan). Secondary antibodies alone (IHC) were included to assess nonspecific binding.

The results of the quantitative analysis included the following: human C/EBPβ immunoreactive score (IRS 0–12 combining intensity and positivity), percentage DAB-positive area for mouse/human markers, and neutrophil cell density (human: CD66b ^+^ within total cells; mouse: CD11b ^+^Ly-6G ^+^ within CD11b ^+^ myeloid cells). Two blinded pathologists performed all analyses using ImageJ (≥ 5 fields/sample). The antibody information is detailed in Supplementary Table S8.

### Cell culture and functional assays

Human colorectal adenocarcinoma Caco-2 and promyelocytic leukemia HL-60 cells (STR-profiled, mycoplasma-free) were obtained from the Institute of Cell Biology, the Chinese Academy of Sciences (Shanghai, China) and cultured using the following protocols. HL-60 cells were differentiated with 5 μM retinoic acid (3 days). Transfections with pcDNA3.1-CEBPB and *CEBPB* siRNA were assessed by qRT-PCR (24 h) and ELISA (CXCL1/2/5; 48 h). Luciferase assays were performed with co-transfected reporter plasmids and β-galactosidase controls (Promega) to investigate the binding of *CEBPB* to the promoters of CXCL1/2/5. For chemotaxis, neutrophil-like HL-60 cells (SB225002-treated) were exposed to Caco-2 supernatants in Transwells (3 μm pores; Corning, Corning, USA), and the number of migrated cells was quantified after crystal violet staining.

### Flow cytometry analysis

Primary colonic immune cells were isolated through sequential enzymatic digestion using EDTA/DTT (Sigma), followed by collagenase D/DNase I/hyaluronidase (Sigma) treatment and Percoll (Sigma) gradient centrifugation [26]. After isolation, the cells were blocked with Fc-specific antibodies and stained with a 7-AAD viability marker prior to analysis with a Attune NxT flow cytometer (Thermo Fisher Scientific) and FlowJo v10. Antibody details are provided in Supplementary Table S8.

### Molecular biology assays

For RNA analysis, total RNA was extracted with TRIzol (Thermo Fisher Scientific), reverse transcribed (TaKaRa, Shiga, Japan), and analyzed by qRT-PCR (StepOnePlus), with *β-actin* used as the endogenous control (sequences are shown in Supplementary Table S9).

For protein analysis: during colonic immune cell isolation, the epithelial and stromal fractions were collected separately. Protein lysates were extracted using RIPA buffer (Beyotime), quantified with a BCA assay kit (Vazyme, Nanjing, China), and analyzed by immunoblotting. Fraction purity was confirmed through marker absence: epithelial fractions showed no detectable CD45 (leukocyte marker) or α-SMA (stromal marker), whereas stromal fractions were negative for Pan-Cytokeratin (epithelial marker).

For chromatin immunoprecipitation (ChIP): DNA-protein complexes were immunoprecipitated using anti-C/EBPβ antibody (ChIP kit; Millipore, Billerica, USA), followed by qPCR analysis of the precipitated DNA.

For electrophoretic mobility shift assay (EMSA): a LightShift Chemiluminescent EMSA kit (Thermo Fisher Scientific) was used for EMSA analysis. Biotin-labelled DNA probes containing putative C/EBPβ binding sites were incubated with recombinant human C/EBPβ protein to form DNA-protein complexes. The reaction mixtures were resolved on 6% non-denaturing polyacrylamide gels in 0.5× TBE buffer (100 V, 60 min), transferred to nylon membranes (380 mA, 30 min) and UV-crosslinked (120 mJ/cm², 1 min) prior to chemiluminescent detection.

For chemokine quantification: tissue supernatant levels of CXCL1 (EK296; Multi Sciences, Hangzhou, China), CXCL2 (EK2142; Multi Sciences), and CXCL5 (EK0919; Boster, Wuhan, China) were measured by ELISA according to the manufacturers’ protocols.

### RNA sequencing analysis

Total RNA was extracted from mid-distal colon tissues using TRIzol reagent (Thermo Fisher Scientific) following the manufacturer’s guidelines. To remove any genomic DNA contamination, the RNA was treated with RNase-free DNase. The cDNA library was prepared and sequenced on a NovaSeq 6000 platform (Illumina, San Diego, USA) by CapitalBio Corporation (Beijing, China). Differential expression analysis was performed via DESeq2 (v1.1), with genes showing a |log2 fold change (FC)| > 1 and adjusted *P* value < 0.05 considered statistically significant. The raw sequencing data have been deposited in the NCBI Gene Expression Omnibus under accession number GSE264497.

### Bioinformatics analysis

We performed integrated analysis of microarray datasets from GEO (GSE87466: 21 normal, 87 UC; GSE37283: 5 normal, 11 CAC) and TCGA-COAD/READ (51 normal, 383 CRC) to identify consistently dysregulated transcription factors in UC, CAC and CRC. All the samples were processed using Affymetrix HT HG-U133 + PM arrays. The data were normalized via robust multiarray average (RMA) and batch-corrected with the ComBat algorithm. Candidate transcription factors were screened via the Animal Transcription Factor Database (animal TFDB v3.0), with differential expression defined as |log2FC| > 1 and *P* < 0.05 (DEseq2). The results were visualized using ggplot2 and pheatmap R packages. Additionally, immune cell infiltration in CRC and CAC tissues was assessed via the CIBERSORTx website ( http://cibersortx.standford.edu/) [ 27, 28]. For functional annotation, we conducted KEGG pathway analysis (DAVID) ( https://david.ncifcrf.gov/) [29] and GSEA via the Broad Institute platform [30]. Statistical significance thresholds were set at *P* < 0.05 for KEGG and FDR < 0.25 for GSEA.

### Statistical analysis

Data are presented as the mean ± standard deviation (SD). All analyses were performed via GraphPad Prism 9.0 (GraphPad Software Inc., La Jolla, USA). Normality was assessed using Shapiro-Wilk tests, whereas homogeneity of variance was evaluated with Brown-Forsythe tests. Parametric tests were applied to normally distributed data with equal variances: unpaired two-tailed *t* tests for two-group comparisons and one-way ANOVA with Bonferroni correction for multiple comparisons. Non-normally distributed data were analyzed via Mann-Whitney U tests (two groups) or Kruskal-Wallis tests with Dunn’s post hoc correction (multiple groups). For normally distributed data with unequal variances, Welch’s *t* test was employed. Linear relationships between biomarkers (C/EBPβ, CXCL1/2/5, and CD66b) were quantified using Pearson correlation coefficients. Statistical significance was defined as *P* < 0.05 (two-tailed), with exact *P* values reported except when *P* < 0.001.

## Results

### Identification of dysregulated transcription factors in UC, CAC, and CRC pathogenesis

**Figure 1:**
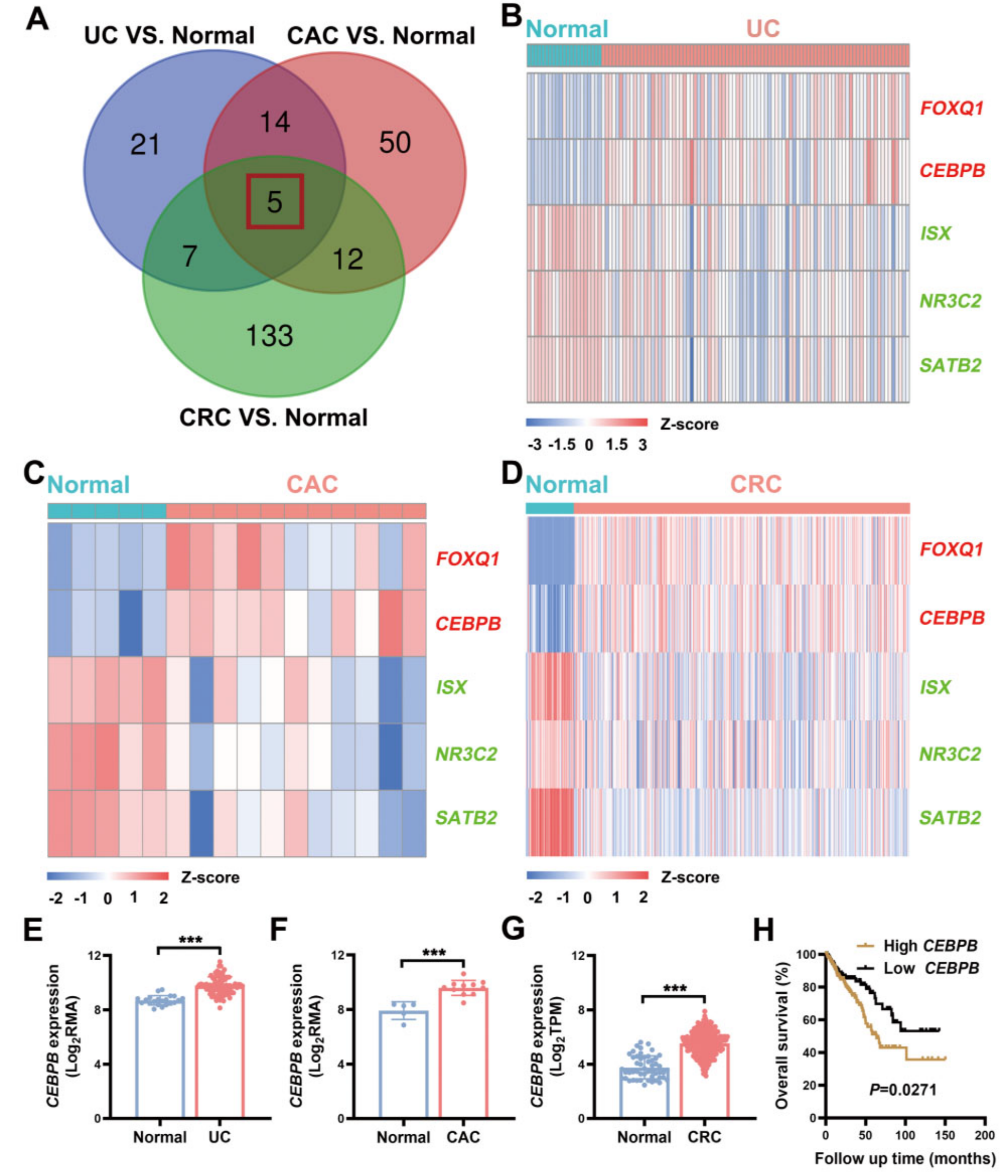
Figure 1 Transcriptional landscape of transcription factors in patients with ulcerative colitis (UC), colitis-associated cancer (CAC), and colorectal cancer (CRC) (A) Venn diagram of differentially expressed transcription factors from the GEO/TCGA database. (B–D) Heatmaps of 5 consistently dysregulated transcription factors (red: upregulated; green: downregulated). (E–G) CEBPB mRNA expression in different patient cohorts: UC patients (GSE87466: 21 healthy individuals vs 87 UC patients; Welch’s two-tailed t test); CAC patients (GSE37283: 5 healthy individuals vs 11 CAC; unpaired t test); and CRC patients [TCGA: 51 normal adjacent tumors (NATs) vs 383 tumors; Mann-Whitney U test]. (H) Kaplan-Meier survival analysis of 376 CRC patients stratified by CEBPB mRNA expression (TCGA). Data are expressed as the mean ± SD. *** P < 0.001.

Through integrated analysis of the GEO and TCGA datasets (Supplementary Tables S1–S3), we identified five transcription factors with consistent dysregulation patterns across UC, CAC, and CRC. Venn analysis comparing UC versus healthy controls, CAC versus controls, and CRC versus normal adjacent tissue (NAT) revealed that *CEBPB* and *FOXQ1* were significantly upregulated (log2FC > 1, *P* < 0.05), whereas *ISX*, *NR3C2*, and *SATB2* were downregulated (log2FC < –1, *P* < 0.05) in all three conditions ( Figure 1A and Supplementary Data S1). Heatmap visualization and histograms confirmed these distinct expression profiles ( Figure 1B–D and Supplemetary Figure S1A–L). *CEBPB* exhibited particularly striking patterns, with progressive overexpression from UC to CAC to CRC ( Figure 1E–G). Clinical correlation analysis demonstrated that elevated *CEBPB* expression significantly predicted poorer survival in CRC patients ( *P <* 0.01; Figure 1H), whereas the other four transcription factors had no prognostic significance ( Supplementary Figure S1M–P). These findings establish CEBPB as a consistently upregulated transcription factor throughout the inflammation-cancer transformation cascade and a potential prognostic marker in CRC.

### C/EBPβ overexpression is correlated with disease progression and poor prognosis in CAC and CRC

**Figure 2:**
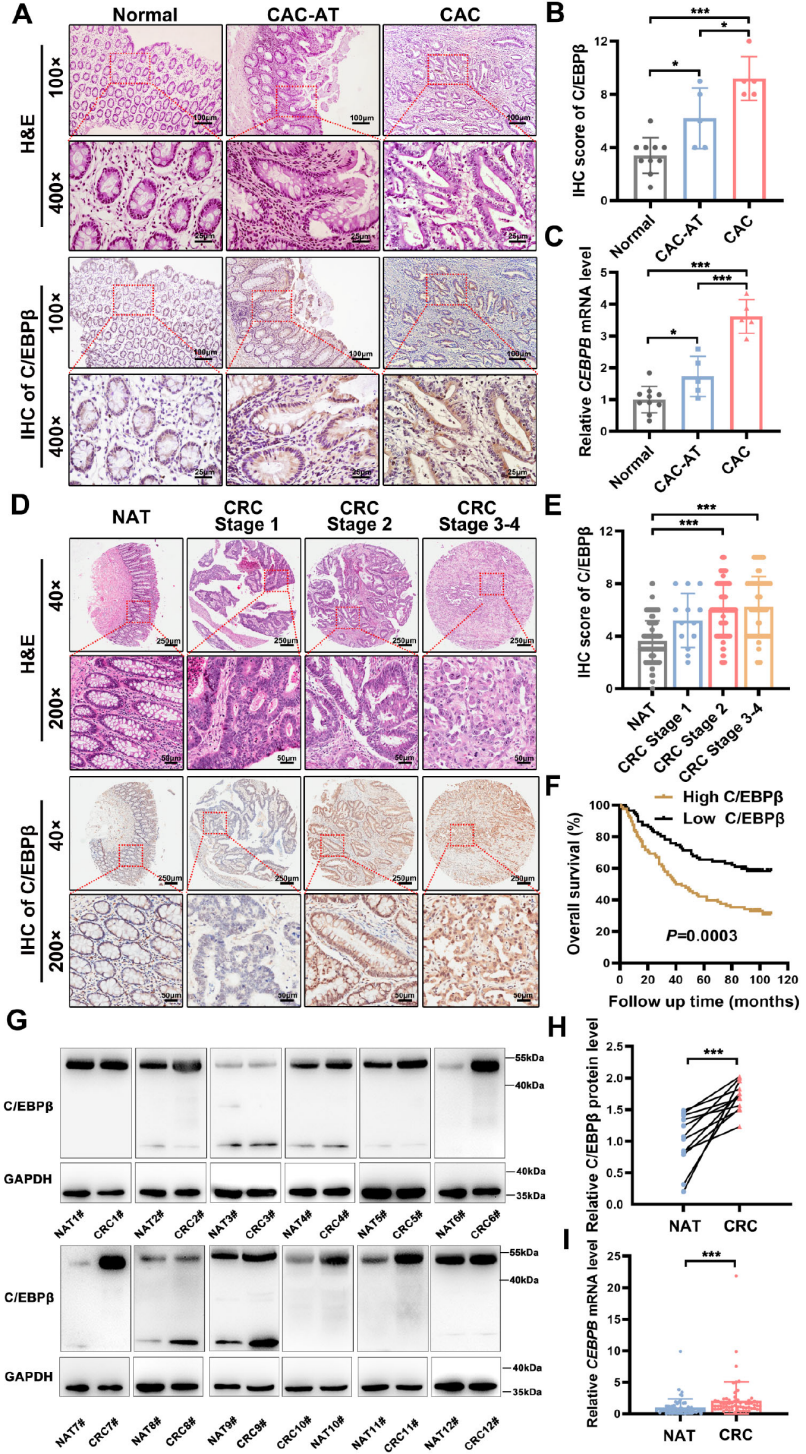
Figure 2 Clinical significance of elevated C/EBPβ in colorectal cancer progression (A) Representative hematoxylin and eosin (H&E) and immunohistochemistry (IHC) staining of C/EBPβ in normal colon, CAC-adjacent (CAC-AT), and CAC tissues (100×: 100 μm scale; 400×: 25 μm scale). (B,C) Quantitative analysis of (B) C/EBPβ protein (IHC) and (C) CEBPB mRNA levels (normal: n = 10; CAC: n = 5; one-way ANOVA with Bonferroni correction). (D) CRC tissue microarrays (n = 180) showing C/EBPβ expression (40×: scale bar = 250 μm; 200×: scale bar = 50 μm). (E) C/EBPβ IHC scores across CRC stages (Kruskal-Wallis test with Dunn’s test). (F) Kaplan-Meier survival analysis of 180 CRC patients stratified by C/EBPβ expression. (G,H) Western blot analysis of C/EBPβ expression in randomly selected CRC patients (n = 12). Protein levels were quantified by densitometry (normalized to the level of GAPDH) using ImageJ software. Statistical analysis was conducted using the Mann-Whitney U test. (I) qRT-PCR was employed to evaluate CEBPB mRNA levels in randomly selected CRC patients (n = 64). Statistical analysis was performed using the Mann-Whitney U test. Data are expressed as the mean ± SD. *P < 0.05, ***P < 0.001.

Immunohistochemical analysis of CAC and UC patient tissues revealed predominant C/EBPβ expression in colonic epithelial cells ( Figure 2A,B, Supplementary Figure S2A,B, and Supplementary Table S5). Quantitative assessment revealed progressively increasing C/EBPβ levels from normal mucosa to adjacent non-cancerous tissues (CAC-AT) and finally to CAC lesions. This increase was confirmed at the transcriptional level by qRT-PCR ( Figure 2C). In sporadic CRC, tissue microarray (TMA) analysis of 180 cases revealed similar C/EBPβ upregulation ( Figure 2D,E and Supplementary Table S4). Importantly, we observed a significant positive correlation between C/EBPβ expression levels and TNM stage ( Figure 2E), with elevated C/EBPβ predicting reduced overall survival ( Figure 2F). Western blot and qRT-PCR validation in independent CRC cohorts ( Supplementary Table S6) confirmed consistent C/EBPβ overexpression ( Figure 2G–I), corroborating our bioinformatics survival analysis ( Figure 1H). These results establish C/EBPβ as a clinically significant biomarker with stage-dependent overexpression and prognostic value in both CAC pathogenesis and sporadic CRC pathogenesis.

### Intestinal epithelial *Cebpb* deletion mitigates tumorigenesis in AOM/DSS-induced murine CAC models

**Figure 3:**
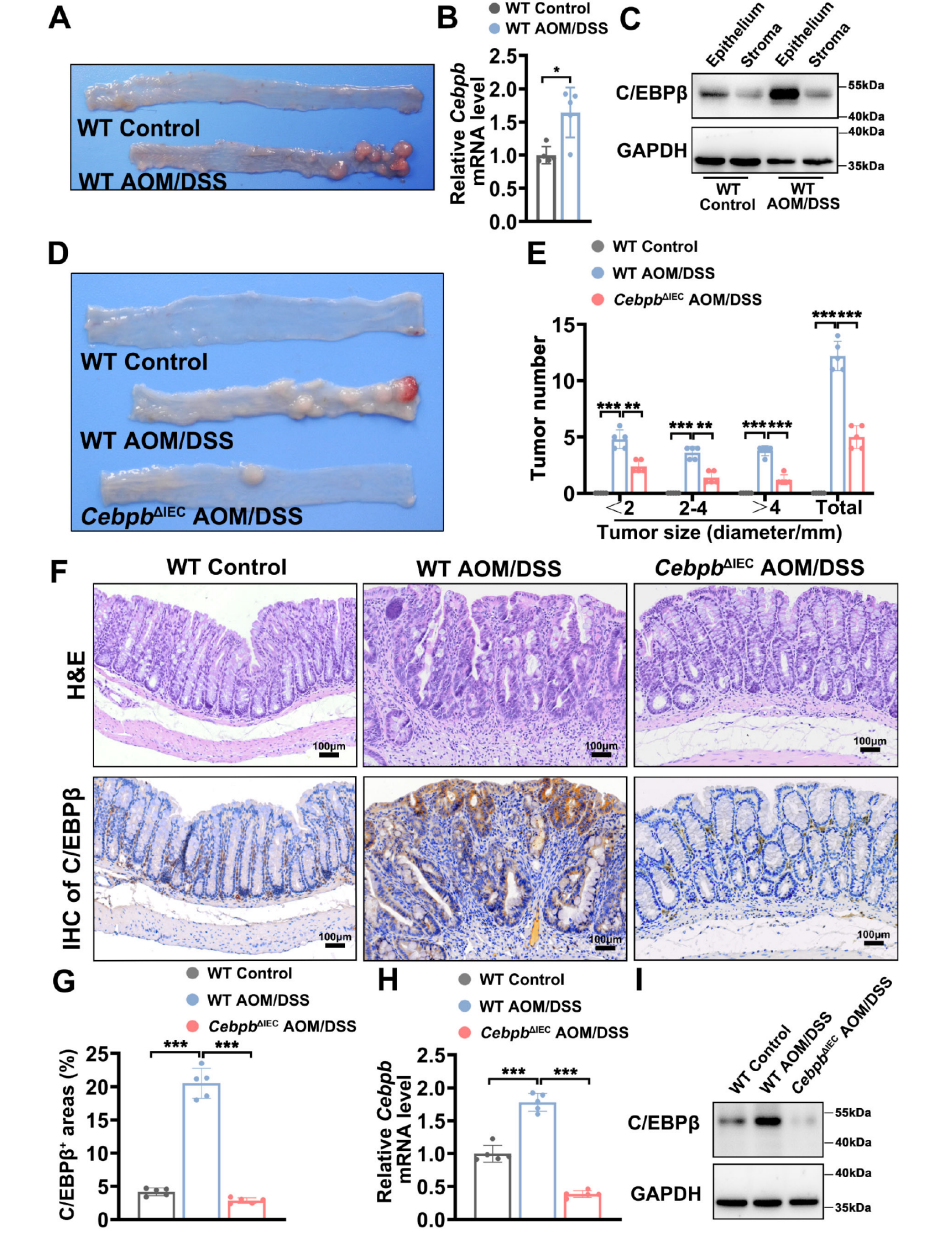
Figure 3 Effects of C/EBPβ deletion in intestinal epithelial cells on tumorigenesis in AOM/DSS-induced murine models (A) Representative images of colons from untreated WT control mice and AOM/DSS-treated WT mice. (B) Cebpb mRNA levels in colonic tissue from WT control and AOM/DSS-treated WT mice were quantified by qRT-PCR. Statistical analysis was performed by Mann-Whitney U test; n = 5 mice/group. (C) Western blot analysis of C/EBPβ protein expression in the colonic epithelium and stroma of WT control and AOM/DSS-treated WT mice. Protein levels were normalized to those of GAPDH. (D) Representative images of colons from three experimental groups: (i) WT control mice, (ii) AOM/DSS-treated WT mice (WT AOM/DSS), and (iii) AOM/DSS-treated mice with intestinal epithelial cell-specific Cebpb knockout (CebpbΔIEC AOM/DSS). Tumor number and size were assessed; n = 5 mice/group. (E) Colon tumors were quantified by size stratification (> 2 mm, measured with callipers;< 2 mm, assessed with a dissection microscope). Data were analyzed by one-way ANOVA with Bonferroni’s multiple comparisons test; n = 5 mice/group. (F) Representative H&E and IHC images of C/EBPβ in colon tissues from WT control, WT AOM/DSS and CebpbΔIEC AOM/DSS mice. Scale bar = 100 μm. (G) Quantification of C/EBPβ-positive staining areas in colon tissues from WT control, WT AOM/DSS and Cebpb ΔIEC AOM/DSS mice by ImageJ software. (H,I) C/ EBPβ mRNA levels (qRT-PCR) and protein expression (western blot analysis) in colon tissues from WT control, WT AOM/DSS and Cebpb ΔIEC AOM/DSS mice. Statistical analysis was performed by Kruskal-Wallis test with Dunn’s multiple comparisons test. n = 5 mice/group for qRT-PCR. Data are presented as the mean ± SD. ns: not significant. * P < 0.05, **P < 0.01, ***P < 0.001.

We successfully established an AOM/DSS-induced CAC model in mice. Representative colon images demonstrate the morphological differences between untreated WT mice and AOM/DSS-treated WT mice ( Figure 3A). Compared with those in control mice, quantitative analysis revealed significantly elevated *Cebpb* mRNA levels in the colonic lesions of AOM/DSS-treated mice ( Figure 3B). Western blot analysis confirmed that this upregulation was specifically localized to intestinal epithelial cells ( Figure 3C), suggesting their central role in C/EBPβ-mediated pathogenesis. To functionally characterize the contribution of C/EBPβ, we generated intestinal epithelial-specific *Cebpb* ^ΔIEC^ mice through Villin-Cre-mediated recombination of floxed alleles ( Supplementary Figure S3). Compared with AOM/DSS-induced littermate control mice, *Cebpb* ^ΔIEC^ mice presented remarkable resistance to AOM/DSS-induced tumorigenesis, with a 62.5% reduction in tumor number ( Figure 3D,E). Histopathological examination revealed that *Cebpb* ^ΔIEC^ mice presented near-normal intestinal architecture with significantly attenuated inflammatory cell infiltration and developed low-grade dysplasia following AOM/DSS treatment ( Figure 3F and Supplementary Table S10). The epithelial-specific deletion of C/EBPβ was verified through immunohistochemical staining ( Figure 3F,G), transcriptional analysis by qRT-PCR ( Figure 3H), and protein level assessment by western blot analysis ( Figure 3I). These findings definitively establish intestinal epithelial C/EBPβ as a critical molecular driver of colitis-associated carcinogenesis.

### Intestinal epithelial C/EBPβ promotes neutrophil recruitment during malignant transformation

**Figure 4:**
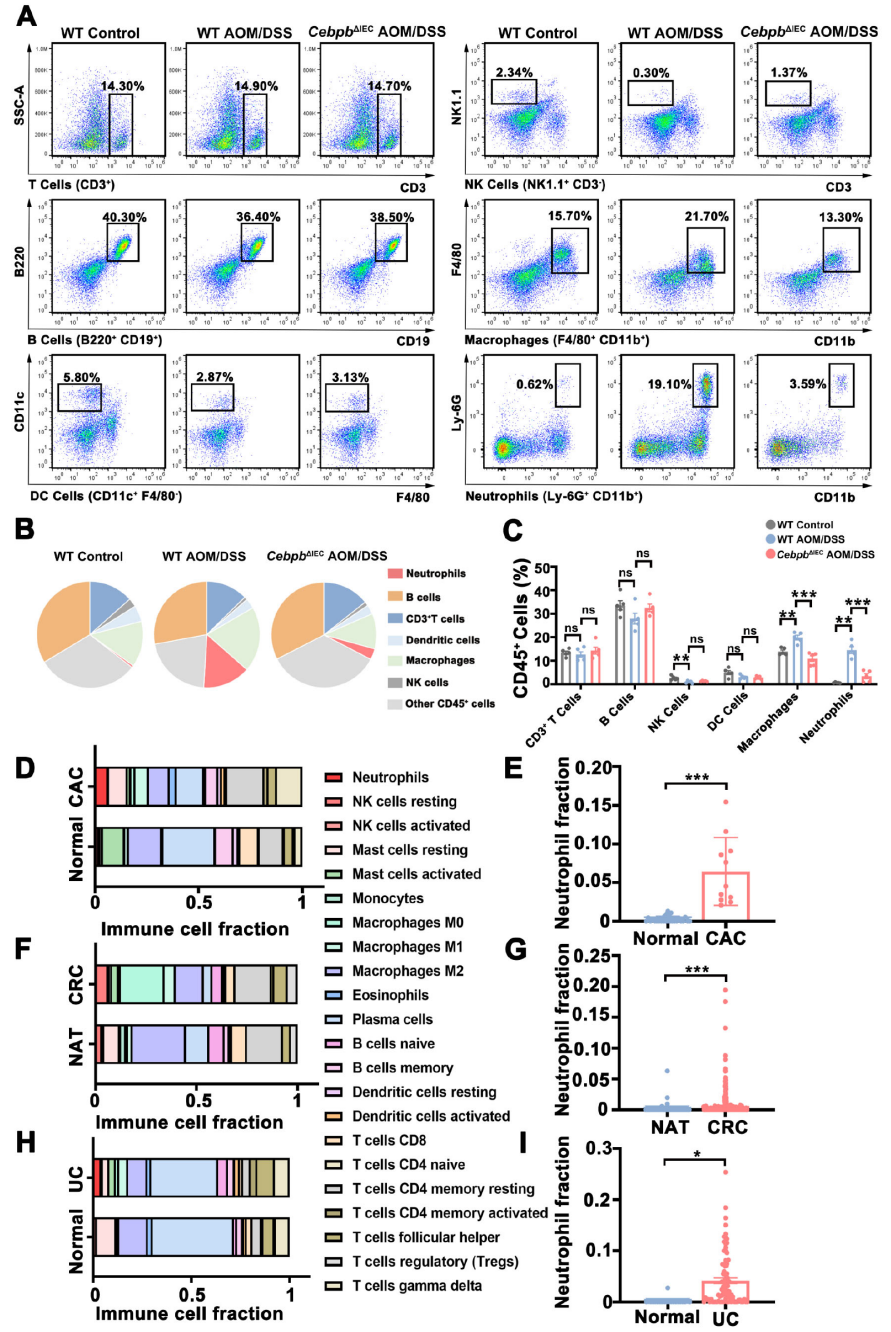
Figure 4 *Cebpb* deletion in intestinal epithelial cells reduces neutrophil infiltration in AOM/DSS-treated mice (A–C) Immune cell subsets in colon tissues from WT control, WT AOM/DSS, and Cebpb ΔIEC AOM/DSS mice were quantified via flow cytometry (n = 3 mice/group). Data were analyzed by one-way ANOVA with Bonferroni’s correction. (D–I) Bioinformatics analysis of neutrophil-associated genes in human datasets (Mann-Whitney U test). For panels (D,E), data were obtained from 26 healthy individuals and 11 CAC patients (GSE87466 and GSE37283). For panels (F,G), data were obtained from 41 normal adjacent tumor tissues (NATs) and 216 CRC samples (TCGA-COAD/READ). For panels (H,I), data were collected from 21 healthy individuals and 87 UC patients (GSE87466 dataset). Data are expressed as the mean ± SD. ns: not significant. *P < 0.05, **P < 0.01, ***P < 0.001.

Flow cytometric analysis of AOM/DSS-treated mice revealed substantial infiltration of innate immune cells, particularly neutrophils and macrophages, in colonic tissues. Targeted deletion of *Cebpb* specifically in intestinal epithelial cells ( *Cebpb* ^ΔIEC^) resulted in a marked 78% reduction in neutrophil recruitment compared with that in AOM/DSS-treated wild-type mice ( Figure 4A–C and Supplementary Figure S4). This selective effect on neutrophil infiltration was further supported by clinical data analysis using the CIBERSORT algorithm, which revealed significantly elevated neutrophil signatures in transcriptomic profiles from patients with UC, CAC, and CRC ( Figure 4D–I). The concordance between murine experimental data and human clinical observations strongly implicates intestinal epithelial C/EBPβ as a specific regulator of neutrophil recruitment during the inflammation-to-cancer transition.

### C/EBPβ transcriptionally activates CXCR2 ligands to mediate neutrophil infiltration in CAC pathogenesis

**Figure 5:**
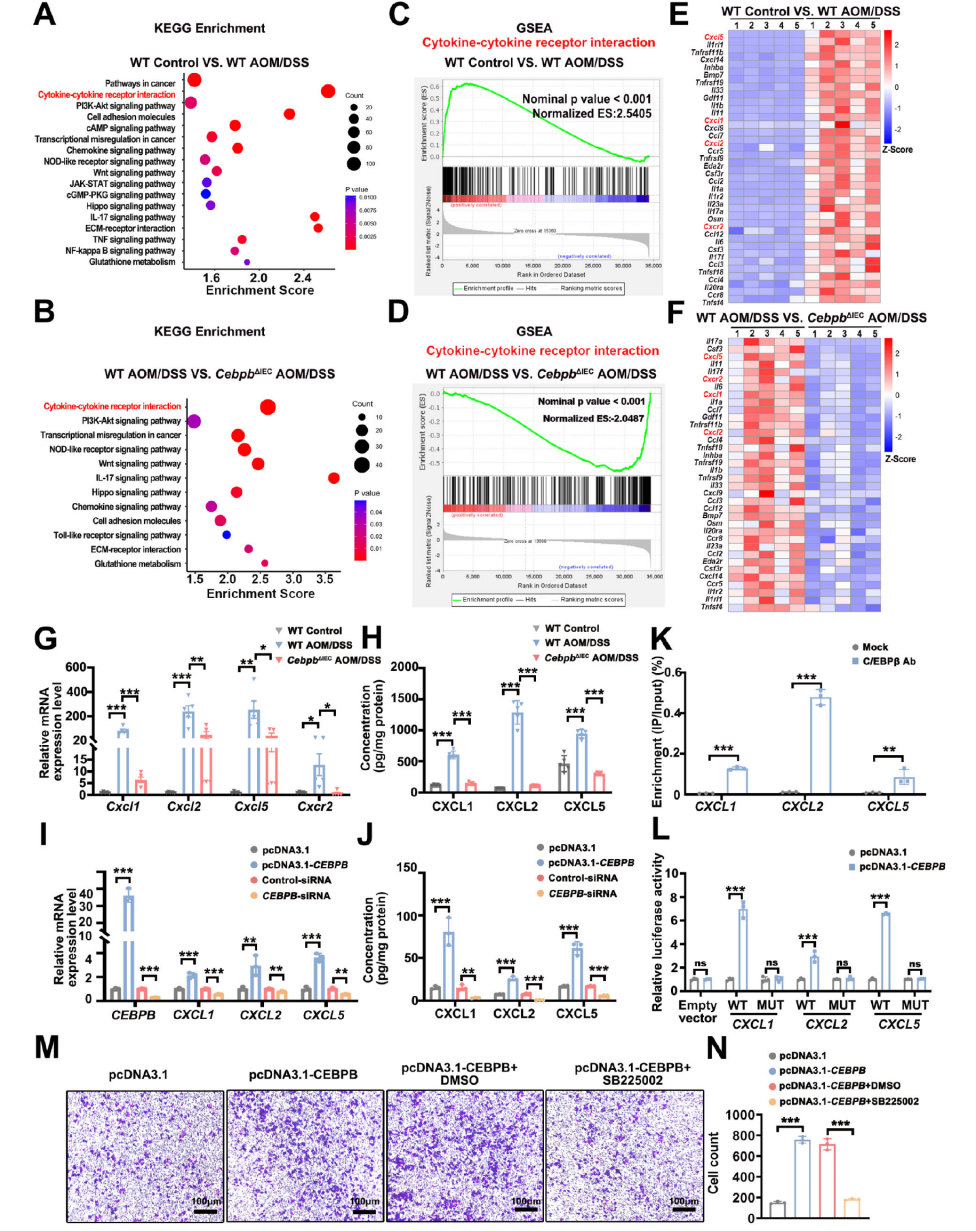
Figure 5 C/EBPβ mediates neutrophil recruitment through the transcriptional upregulation of CXCR2 ligands (A–D) KEGG and gene set enrichment analysis (GSEA) of differentially expressed genes (DEGs) in colon tissues from WT control, WT AOM/DSS, and CebpbΔIEC AOM/DSS mice (n = 5 mice/group). (E,F) Heatmap showing 36 significantly enriched DEGs from the cytokine-cytokine receptor interaction pathway (Z scores are shown in a color gradient). (G,H) CXCR2 ligand (CXCL1/2/5) and receptor levels in colon tissues were measured by qRT-PCR (G; n = 5) and ELISA (H; n = 5). Statistical analysis was performed by one-way ANOVA with Bonferroni’s correction. (I,J) CXCL1/2/5 expression in Caco-2 cells with CEBPB overexpression (pcDNA3.1-CEBPB) or knockdown ( CEBPB-siRNA) compared with their respective controls (one-way ANOVA with Bonferroni’s correction). (K) ChIP-qPCR confirming C/EBPβ binding to the CXCL1/2/5 promoters (anti-C/EBPβ antibody vs input/IgG controls; unpaired t test). (L) Luciferase reporter assays of CEBPB-responsive promoter activity (wild-type vs mutant CXCL1/2/5 promoters) in CEBPB-overexpressing Caco-2 cells (unpaired t test). (M,N) Representative images showing neutrophil migration toward supernatants from CEBPB-modulated Caco-2 cells (SB225002-pretreated; scale bar = 100 μm; one-way ANOVA with Bonferroni’s correction). n = 3 biological replicates for panels I--N. Data are expressed as the mean ± SD. ns: not significant. * P < 0.05, **P < 0.01, ***P < 0.001.

Transcriptomic profiling of colonic tissues from WT, AOM/DSS-treated, and *Cebpb* ^ΔIEC^ AOM/DSS mice revealed significant enrichment of cytokine-cytokine receptor interaction pathways ( Figure 5A,B and Supplementary Data S2 and S3). Gene set enrichment analysis (GSEA) confirmed a strong positive correlation of this pathway in WT AOM/DSS mice versus controls, whereas *Cebpb* deletion reversed this trend ( Figure 5C,D). Among the 36 differentially expressed genes (DEGs) identified by intersection analysis ( Supplementary Figure S5 and Supplementary Table S11), CXCR2 ligands ( *Cxcl1*, *Cxcl2*, and *Cxcl5*) and *Cxcr2* itself were markedly upregulated in WT AOM/DSS mice but significantly attenuated in C *ebpb* ^ΔIEC^ mice ( Figure 5E,F). This regulation was validated at both the transcriptional (qRT-PCR) and protein (ELISA) levels ( Figure 5G,H). The leading edge subset of genes is detailed in Supplementary Data S4.

Mechanistic studies in Caco-2 cells demonstrated that C/EBPβ directly activates CXCL1/2/5 transcription. Overexpression of C/EBPβ increased CXCL1/2/5 expression, whereas its knockdown reduced CXCL1/2/5 expression ( Figure 5I,J). Our integrated computational (JASPAR) and experimental (ChIP-qPCR) analyses identified C/EBPβ binding sites in the promoter regions of CXCL1 (chr4: 73868520-73868724), CXCL2 (chr4 complement: 74100928-74100673), and CXCL5 (chr4 complement: 74000013-73999933) ( Figure 5K). Functional validation through luciferase reporter assays confirmed that mutation of these sites abolished C/EBPβ-mediated transactivation ( Figure 5L), whereas EMSA demonstrated direct C/EBPβ binding *in vitro* ( Supplementary Figure S6). Using *CXCR2*-deficient Caco-2 cells [31], we further showed that supernatants from C/EBPβ-overexpressing cultures induced robust neutrophil chemotaxis, an effect potently inhibited by the selective CXCR2 antagonist SB225002 ( Figure 5M,N) [32]. These results establish a C/EBPβ→CXCL1/2/5→CXCR2 signaling axis whereby intestinal epithelial C/EBPβ transcriptionally drives neutrophil recruitment during colitis-associated carcinogenesis.

### Pharmacological blockade of the C/EBPβ-CXCR2 axis attenuates neutrophil-driven tumorigenesis in CAC

**Figure 6:**
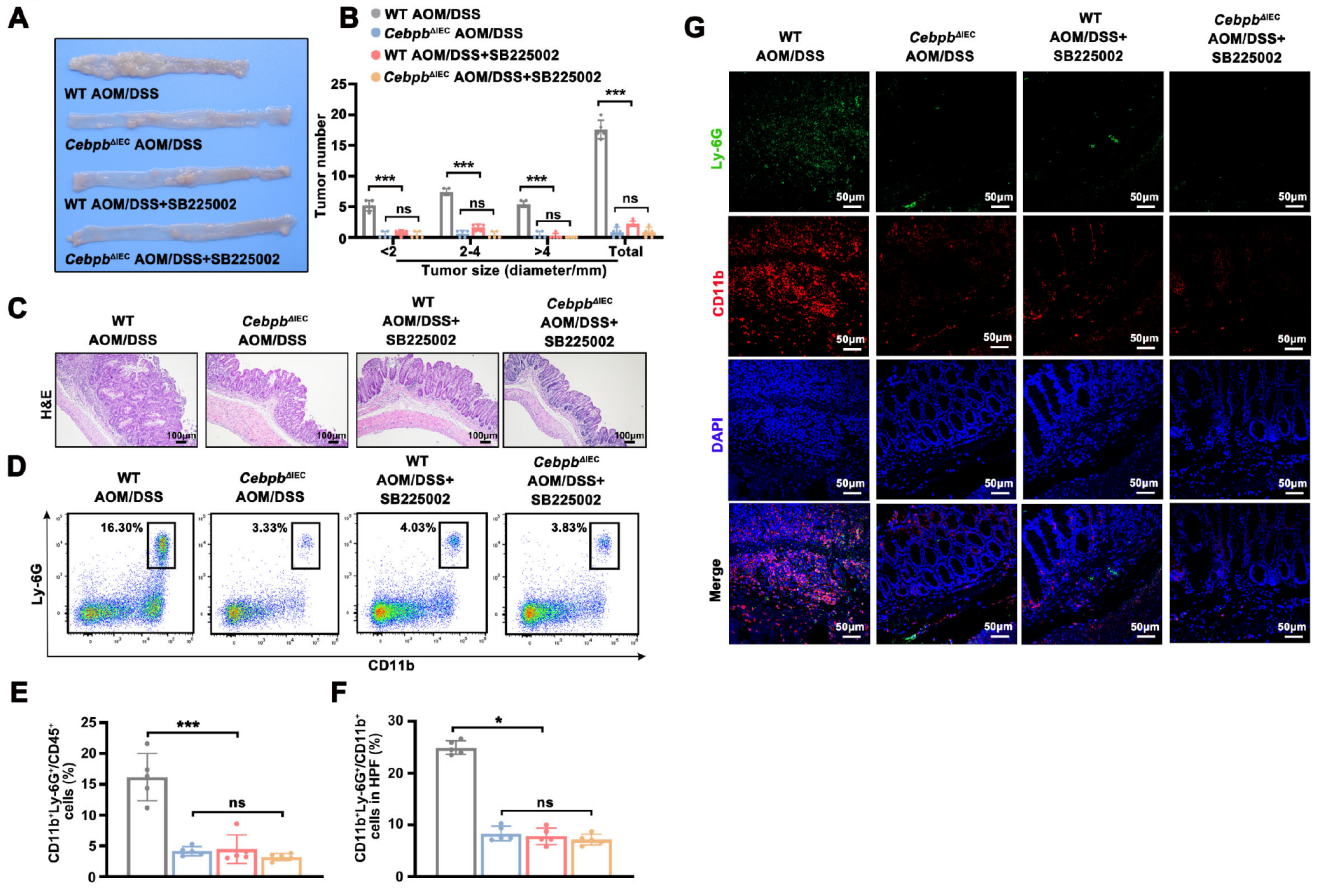
Figure 6 Pharmacological blockade of the C/EBPβ-CXCR2 axis reduces neutrophilic inflammation and tumorigenesis in the AOM/DSS-induced CAC model (A) Colony samples were harvested from four groups: (i) WT AOM/DSS, (ii) CebpbΔIEC AOM/DSS, and (iii–iv) SB225002 (CXCR2 antagonist)-treated counterparts (WT or CebpbΔIEC AOM/DSS mice). (B) Tumor burden quantification (> 2 mm by callipers; < 2 mm by dissection microscopy; one-way ANOVA with Bonferroni’s correction). n = 5 mice/group. (C) Representative H&E staining of colon tissues from the four groups (scale bar = 100 μm). (D,E) Flow cytometry analysis of CD11b+Ly-6G+ neutrophils among CD45+ leukocytes (one-way ANOVA with Bonferroni’s correction). (F,G) Immunofluorescence visualization (scale bar = 50 μm) and ImageJ quantification of CD11b+Ly-6G+ cell infiltration in 5 high-power fields (HPFs). n = 5 mice/group. Data are expressed as the mean ± SD. ns: not significant. *P < 0.05, ***P < 0.001.

Building on previous reports demonstrating that neutrophil depletion reduces AOM/DSS-induced tumor burden [32], we investigated whether targeting the C/EBPβ-CXCR2 axis could similarly impair carcinogenesis. The CXCR2 antagonist SB225002 significantly reduced the tumor burden in AOM/DSS-treated mice, as evidenced by decreased tumor number, size and histologic grade ( Figure 6A,B and Supplementary Table S12), recapitulating the protective effects observed in *Cebpb* ^ΔIEC^ mice. Flow cytometric and immunofluorescence analysis confirmed that, compared with genetic Cebpb ablation, SB225002 treatment reduced colonic neutrophil infiltration ( Figure 6C–G). Notably, combined SB225002 treatment and *Cebpb* deletion provided no additional benefit, indicating that both interventions target the same pathway. These findings demonstrate that intestinal epithelial C/EBPβ promotes CAC progression predominantly through transcriptional activation of CXCR2 ligands (CXCL1/2/5), establishing this axis as a central mechanism for neutrophil recruitment and subsequent tumor growth in colitis-associated carcinogenesis.

### Immunohistochemical validation and correlation analysis of C/EBPβ-driven neutrophil infiltration in clinical samples

**Figure 7:**
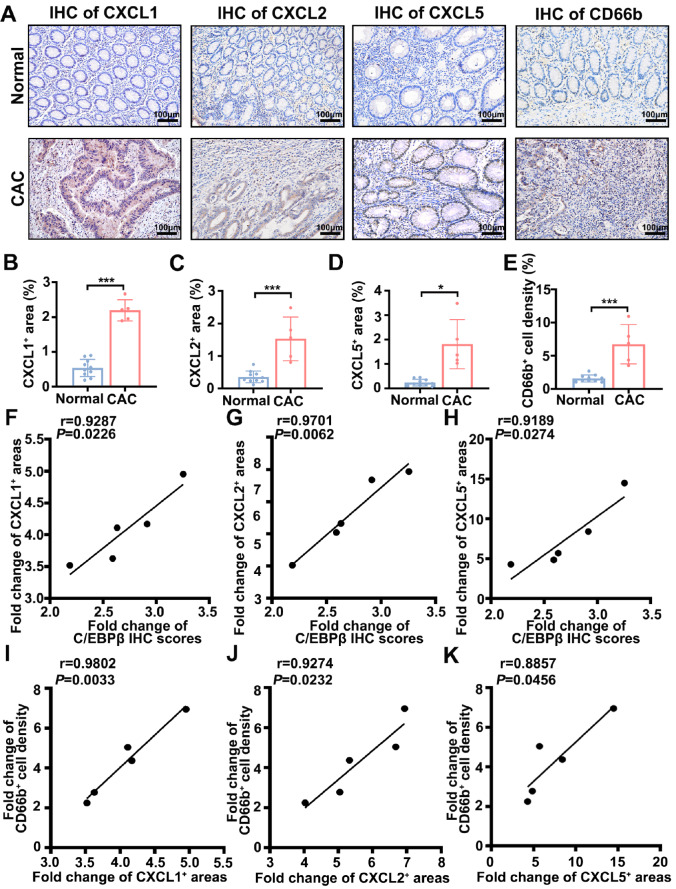
Figure 7 Clinical validation of C/EBPβ-mediated neutrophil recruitment in CAC (A) Representative IHC staining of CXCL1/2/5 and the neutrophil marker CD66b in normal colons ( n = 10) vs CAC (n = 5) (scale bar = 100 μm). (B–E) Quantification of chemokine staining areas and CD66b+ cell density (CXCL1: unpaired t test; CXCL2/5/CD66b: Welch’s t test). (F–K) Pearson correlation analysis: (F–H) C/EBPβ IHC scores vs CXCL1/2/5+ areas; (I–K) CD66b+ cell density vs CXCL1/2/5 + areas. The Pearson correlation coefficient (r) is indicated. Data are expressed as the mean ± SD. *P < 0.05, ***P < 0.001.

Immunohistochemical analysis of clinical samples revealed significantly elevated expression of CXCR2 ligands (CXCL1/2/5) and increased neutrophil infiltration (CD66b ^+^) in CAC and CRC tissues compared with healthy controls or NATs ( Figure 7A–E and Supplementary Figure S7A–E). Strikingly, C/EBPβ levels exhibited a robust positive correlation with CXCR2 ligands, whereas CXCL1/2/5 expression further correlated with neutrophil infiltration in both the CAC and CRC cohorts ( Figure 7F–K and Supplementary Figure S7F–K). These results collectively demonstrate the critical role of the C/EBPβ-CXCL1/2/5 axis in driving neutrophil infiltration during CAC pathogenesis.

## Discussion

Our study established intestinal epithelial C/EBPβ as a key transcriptional regulator linking chronic inflammation to colitis-associated carcinogenesis. Through integrated multi-omics analysis, we identified consistent C/EBPβ upregulation across the UC-CAC-CRC continuum, with elevated expression correlating with poorer clinical outcomes. Genetic ablation studies confirmed its functional importance, as epithelial-specific *Cebpb* deletion markedly attenuated both inflammatory responses and tumor development in AOM/DSS models. Mechanistically, we demonstrated that C/EBPβ orchestrates neutrophil recruitment through direct transcriptional activation of CXCR2 ligands (CXCL1/2/5), creating a pro-tumorigenic microenvironment.

Our transcriptomic profiling revealed four additional transcription factors with distinct roles in CAC pathogenesis. FOXQ1 was upregulated in our models, which is consistent with its known pro-tumorigenic roles in CRC through angiogenic and anti-apoptotic pathways [ 32– 34]. NR3C2 was significantly downregulated, aligning with its tumor suppressive function via AKT/ERK modulation [ 35, 36]. The specific contributions of FOXQ1 and NR3C2 to CAC require further elucidation. SATB2 depletion, which is associated with poor prognosis in CRC [ 37– 39], may aggravate CAC through impaired ion homeostasis and microbial dysbiosis [ 40, 41]. ISX is a novel candidate that requires functional characterization in CAC. These findings position C/EBPβ at the core of a broader transcriptional network governing inflammation-driven carcinogenesis.

C/EBPβ is well established as a key regulator of immune responses, controlling immune cell development and inflammatory cytokine production [ 42– 44]. Although previous studies have characterized its role in circulating immune cells during IBD [ 45, 46], our study revealed its critical epithelial-specific functions in inflammation-driven carcinogenesis. We demonstrated that epithelial C/EBPβ governs neutrophil recruitment to colonic tumors through direct regulation of CXCL1/2/5 expression, as confirmed by ChIP-qPCR, luciferase assays and EMSA. This epithelial-immune crosstalk is clinically relevant, as evidenced by: (i) a correlation with neutrophil accumulation in human CAC/CRC specimens, as previously reported [46]; and (ii) a phenocopy of the *Cebpb* ^ΔIEC^ phenotype resulting from CXCR2 inhibition (SB225002). Notably, the non-additive effect of SB225002 in *Cebpb* ^ΔIEC^ mice confirms pathway specificity, which is consistent with therapeutic outcomes from neutrophil depletion using an anti-Gr-1 antibody in the AOM/DSS model [47]. While IL-8 is a well-established transcriptional target of C/EBPβ in humans and binds to CXCR2 to drive neutrophil recruitment, mice lack an IL-8 orthologue [ 48, 49]. Instead, CXCR2 activation in the AOM/DSS model is mediated by the functional analogues CXCL1/2/5 [50], establishing a C/EBPβ→CXCL1/2/5→CXCR2→neutrophil recruitment cascade that explains the therapeutic synergy between genetic and pharmacological interventions. Our *in vitro* chemotaxis assays confirmed that SB225002 potently inhibited C/EBPβ-driven neutrophil migration, demonstrating functional conservation of this pathway despite species-specific ligand differences. These findings reveal an epithelial-specific role for C/EBPβ in CAC through CXCR2 ligand-dependent mechanisms, distinct from its immune cell functions [ 44, 45], suggesting tissue-specific therapeutic targeting strategies.

In addition to regulating neutrophil recruitment through the CXCL1/2/5-CXCR2 axis, our study further demonstrated that C/EBPβ coordinates multiple pro-tumorigenic pathways in colitis-associated carcinogenesis. Genetic ablation of C/EBPβ significantly reduced the levels of key inflammatory cytokines (IL-1β, IL-6, IL-11, IL-17A, and CCL2; Supplementary Figure S4), revealing its broader control of tumor-promoting inflammation. Mechanistically, IL-11 is known to promote the inflammation-cancer transition through STAT3 activation in epithelial/stromal cells [51], IL-17A contributes to colitis-associated tumorigenesis despite its immune origin [52], and CCL2 plays a pivotal role in recruiting tumor-promoting macrophages [53], with its downregulation explaining the reduced macrophage infiltration in *Cebpb* ^ΔIEC^ mice. Collectively, these findings position C/EBPβ as a master regulator of epithelial-immune crosstalk in CAC. The conserved mechanism across murine models and human diseases and the druggable nature of multiple downstream effectors (CXCR2 ligands, IL-11/STAT3, and CCL2) highlight promising therapeutic opportunities, particularly through combinatorial targeting strategies or direct epithelial C/EBPβ inhibition for CAC treatment. Our work provides both mechanistic insights for understanding inflammation-driven tumorigenesis and actionable targets for CAC intervention.

## Supporting information

25384Supplementary_Data

## Supplementary Data

Supplementary data are available at *Acta Biochimica et Biophysica Sinica* online.
